# *Vital Signs:* Hepatitis C Treatment Among Insured Adults — United States, 2019–2020

**DOI:** 10.15585/mmwr.mm7132e1

**Published:** 2022-08-12

**Authors:** William W. Thompson, Hasan Symum, Amy Sandul, DHSc, Neil Gupta, Priti Patel, Noele Nelson, Jonathan Mermin, Carolyn Wester

**Affiliations:** ^1^Division of Viral Hepatitis, National Center for HIV, Viral Hepatitis, STD, and TB Prevention, CDC; ^2^Office of the Director, National Center for HIV, Viral Hepatitis, STD, and TB Prevention, CDC.

## Abstract

**Introduction:**

Over 2 million adults in the United States have hepatitis C virus (HCV) infection, and it contributes to approximately 14,000 deaths a year. Eight to 12 weeks of highly effective direct-acting antiviral (DAA) treatment, which can cure ≥95% of cases, is recommended for persons with hepatitis C.

**Methods:**

Data from HealthVerity, an administrative claims and encounters database, were used to construct a cohort of adults aged 18–69 years with HCV infection diagnosed during January 30, 2019–October 31, 2020, who were continuously enrolled in insurance for ≥60 days before and ≥360 days after diagnosis (47,687). Multivariable logistic regression was used to assess the association between initiation of DAA treatment and sex, age, race, payor, and Medicaid restriction status; adjusted odds ratios (aORs) and 95% CIs were calculated.

**Results:**

The prevalence of DAA treatment initiation within 360 days of the first positive HCV RNA test result among Medicaid, Medicare, and private insurance recipients was 23%, 28%, and 35%, respectively; among those treated, 75%, 77%, and 84%, respectively, initiated treatment within 180 days of diagnosis. Adjusted odds of treatment initiation were lower among those with Medicaid (aOR = 0.54; 95% CI = 0.51–0.57) and Medicare (aOR = 0.62; 95% CI = 0.56–0.68) than among those with private insurance. After adjusting for insurance type, treatment initiation was lowest among adults aged 18–29 and 30–39 years with Medicaid or private insurance, compared with those aged 50–59 years. Among Medicaid recipients, lower odds of treatment initiation were found among persons in states with Medicaid treatment restrictions (aOR = 0.77; 95% CI = 0.74–0.81) than among those in states without restrictions, and among persons whose race was coded as Black or African American (Black) (aOR = 0.93; 95% CI = 0.88–0.99) or other race (aOR = 0.73; 95% CI = 0.62–0.88) than those whose race was coded as White.

**Conclusions and Implications for Public Health Practice:**

Few insured persons with diagnosed hepatitis C receive timely DAA treatment, and disparities in treatment exist. Unrestricted access to timely DAA treatment is critical to reducing viral hepatitis–related mortality, disparities, and transmission. Treatment saves lives, prevents transmission, and is cost saving.

## Introduction

Despite the availability of accurate diagnostic tests and an effective cure, approximately 2.2 million civilian, noninstutionalized adults had hepatitis C virus (HCV) infection in the United States during January 2017–March 2020,^†^ and incidence continues to rise, particularly among younger adults and in association with injection drug use (*1*,*2*). Untreated, hepatitis C can lead to advanced liver disease, liver cancer, and death (*3*). Hepatitis C treatment with direct-acting antiviral (DAA) agents is recommended for all persons with HCV infection with few exceptions (e.g., persons with a very limited life expectancy and children aged <3 years) (*4*).

Hepatitis C treatment saves lives, prevents transmission, and is cost saving (*5*–*8*). Short course, safe, well-tolerated, oral-only hepatitis C treatment results in a cure in ≥95% of cases (*9*). However, only an estimated 1.2 million persons initiated hepatitis C treatment with DAA agents in the United States during 2014–2020 (*10*), far below the number needed to achieve national hepatitis C elimination goals (*11*). Further, the number of persons treated was highest in 2015 and declined to its lowest level in 2020 (*10*); approximately 14,200 hepatitis C–related deaths were reported in the United States in 2019 (*2*). This analysis used a large national health care claims database to assess hepatitis C treatment among persons with diagnosed HCV infection by sex, age, race, insurance type (i.e., private, Medicaid, and Medicare), and by state Medicaid treatment restrictions.

## Methods

Deidentified data came from HealthVerity, a nationwide administrative claims and encounters database containing longitudinal person-level enrollment records, laboratory test results, and prescription information.^§^ The retrospective cohort in this study included approximately 2 million persons from all 50 states and the District of Columbia enrolled in private insurance plans, Medicare Advantage, or Medicaid managed care who had received a test for HCV infection and had ≥1 day of enrollment in either private insurance, Medicaid, or Medicare coverage (Supplementary Table, https://stacks.cdc.gov/view/cdc/119619). HealthVerity claims capture complete health care use and enrollment records across physician outpatient visits, diagnostic centers, and pharmacies. Enrollment, laboratory test, and pharmacy claims databases were linked using HealthVerity’s person-level deterministic proprietary matching algorithm.

An analytic cohort of patients with hepatitis C (those who received at least one positive HCV RNA test result during January 30, 2019–October 31, 2020) was created by selecting from among patients aged 18–69 years who received any HCV test. The earliest date of receipt of a positive HCV RNA test result that occurred within the selected time frame was defined as the index HCV RNA–positive test date. Eligible persons had continuous enrollment in medical and pharmacy plans for ≥60 days before and ≥360 days after the index RNA-positive test date, and no evidence of DAA treatment during the 60 days preceding the index HCV RNA test date. Initiation of DAA treatment was defined as receipt of any prescription using the Food and Drug Administration and American Association for the Study of Liver Diseases/Infectious Diseases Society of America National Drug Codes definition.^¶^ For persons with a DAA treatment pharmacy claim, the first DAA prescription date was assigned as the index DAA treatment date. The interval from the positive index RNA test result to DAA treatment date for the treatment cohort was defined as the difference between the index HCV RNA–positive test date and the index DAA prescription fill date. Initiation of DAA treatment prevalence was calculated as the percentage of eligible patients who initiated DAA treatment within 360 days of the index RNA-positive test date. The primary outcome for analysis was receipt of a DAA pharmacy claim during the 360-day follow-up period. Covariates included sex (i.e., female or male), age group (i.e., 18–29, 30–39, 40–49, 50–59, and 60–69 years), race (i.e., White, Black, Asian, or other race), and insurance type (i.e., private, Medicaid managed care, and Medicare Advantage). Ethnicity was only available for 39% of persons and was not included in the primary analyses. Medicaid treatment restrictions were defined as state Medicaid programs imposing any of three restrictions before authorization of DAA treatment: presence of liver fibrosis meeting fibrosis stage criteria, mandated sobriety or abstinence from alcohol or drugs (≥1 month), or requirement for prescription by or in consultation with a specialist. State-level Medicaid treatment restrictions data were obtained from HepVu,** an online platform used to visualize data and disseminate information on the U.S. hepatitis epidemic. State-level restriction was defined as the presence of one or more restrictions at the time of patient index HCV RNA–positive test date. Data were excluded from this analysis for persons who had positive HCV RNA test results but were missing sex, age, or state of residence (0.4%).

DAA treatment initiation was assessed using point estimates and 95% CIs; a Wald chi-square test of independence was used to compare baseline characteristics by treatment status. Multivariable logistic regression models were used to quantify the association between the covariates and HCV DAA treatment, adjusting for sex, age group, race, insurance type, and Medicaid treatment restrictions status; aORs and 95% CIs were calculated with p<0.05 considered statistically significant. Sensitivity analyses were conducted to assess potential effects of missing ethnicity data, alternative codings for race, and impact of state Medicaid treatment restrictions. Analyses were conducted using Azure Databricks (web version; Databricks) and RStudio (version 4.1; RStudio). This activity was reviewed by CDC and conducted consistent with applicable federal law and CDC policy.^††^

## Results

**FIGURE 1:**
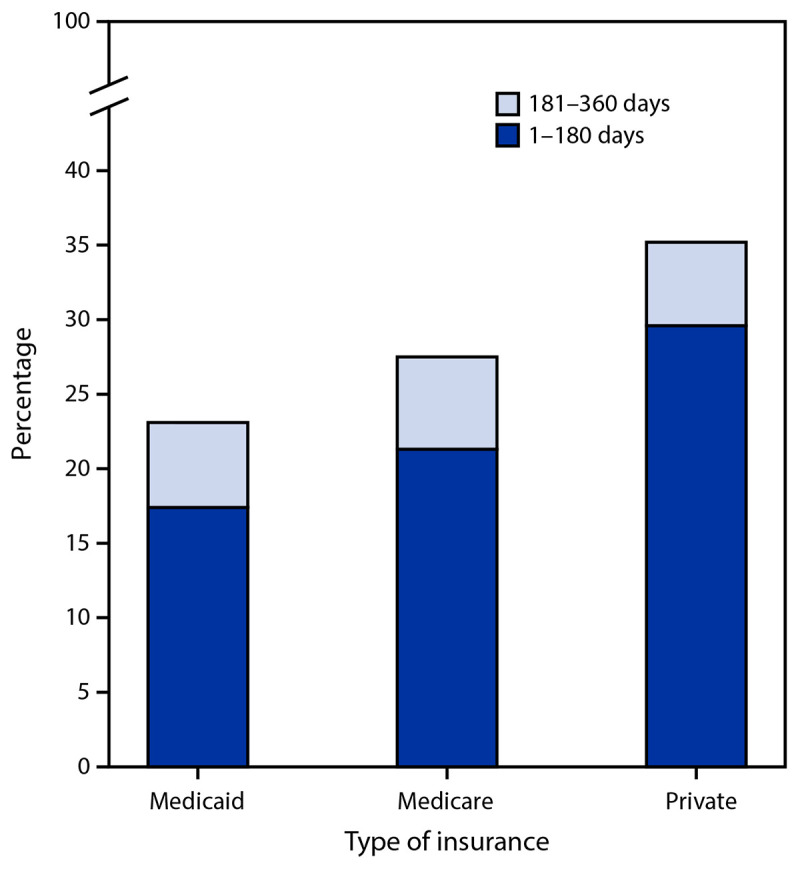
Percentage of adults with hepatitis C initiating direct-acting antiviral treatment within 360 days of diagnosis, by number of days after diagnosis and insurance type — United States, 2019–2020

During January 30, 2019–October 31, 2020, among 81,913 persons who had at least one positive HCV RNA test result, 47,687 (58%) met inclusion criteria (Supplementary Table, https://stacks.cdc.gov/view/cdc/119619). Medicaid managed care covered 37,877 (79%) persons who had a positive HCV RNA test result (Table 1). DAA treatment initiation within 360 days of receipt of a positive HCV RNA test result among persons continuously enrolled in Medicaid, Medicare, and private insurance was 23%, 28%, and 35%, respectively (Figure 1). Among patients who received treatment, 84% of private insurance recipients initiated DAA treatment within 180 days of index HCV RNA–positive test date, compared with 75% of Medicaid and 77% of Medicare recipients.

**TABLE 1:** Characteristics of patients with hepatitis C,* by insurance provider — HealthVerity, United States, 2019–2020^†^

Characteristic	Medicaid^§^		Medicare^¶^		Private	
	No. of unique patients with HCV RNA test^†^	No. (%) with positive HCV RNA test result**	No. of unique patients with HCV RNA test^†^	No. (%) with positive HCV RNA test result**	No. of unique patients with HCV RNA test^†^	No. (%) with positive HCV RNA test result**
**Total**	**88,490**	**37,877 (42.8)**	**11,583**	**3,218 (27.8)**	**32,559**	**6,592 (20.2)**
**Sex**						
Female	42,585	15,812 (37.1)	4,842	1,177 (24.3)	15,270	2,384 (15.6)
Male	45,905	22,065 (48.1)	6,741	2,041 (30.3)	17,289	4,208 (24.3)
**Age group, yrs**						
18–29	13,735	5,690 (41.4)	97	28 (28.9)	3,918	722 (18.4)
30–39	21,734	10,674 (49.1)	449	174 (38.8)	5,208	1,140 (21.9)
40–49	14,961	6,683 (44.7)	816	269 (33.0)	5,114	1,041 (20.4)
50–59	22,335	8,909 (39.9)	2,536	696 (27.4)	9,193	1,831 (19.9)
60–69	15,725	5,921 (37.7)	7,685	2,051 (26.7)	9,096	1,858 (20.4)
**Race**						
White	54,009	24,374 (45.1)	6,417	1,778 (27.7)	15,378	3,276 (21.3)
Black	19,346	7,666 (39.6)	3,164	879 (27.8)	5,817	1,169 (20.1)
Asian	2,651	934 (35.2)	317	95 (30.0)	1,131	151 (13.4)
Other	2,297	841 (36.6)	281	72 (25.6)	2,059	383 (18.6)
Missing	10,187	4,062 (39.9)	1,404	394 (28.1)	8,174	1,613 (19.7)
**State Medicaid treatment restrictions^††^**						
No	44,239	17,083 (38.8)	—	—	—	—
Yes^§§^	44,251	20,794 (47.0)	—	—	—	—

**Abbreviation:** HCV = hepatitis C virus.

* Persons with hepatitis C are patients with a positive HCV RNA test result.

^†^ Continuous enrollment in medical and pharmacy plans for ≥60 days before and ≥360 days after the RNA-positive index date during January 30, 2019–October 31, 2020.

^§^ Medicaid managed care.

^¶^ Medicare Advantage programs.

** Data spans December 1, 2018–October 31, 2021. Continuous enrollment in medical and pharmacy plans for ≥60 days before and ≥360 days after the RNA-positive index date during January 30, 2019–October 31, 2020.

^††^ Data restricted to Medicaid recipients only.

^§§^ Living in a state with a Medicaid liver fibrosis or sobriety requirement (≥1 month of abstinence from alcohol or drugs) or prescriber restriction.

**FIGURE 2:**
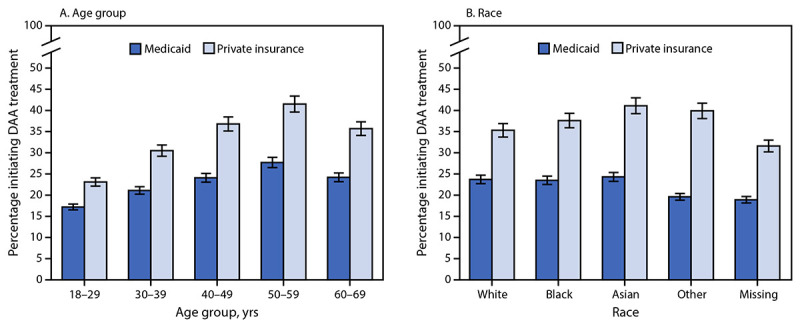
Percentage of adults* with hepatitis C initiating direct-acting antiviral treatment, by insurance type, age group (A), and race (B) — United States, 2019–2020 **Abbreviation:** DAA = direct-acting antiviral. * With 95% CIs shown by error bars.

Comparison of DAA treatment initiation by age group and insurance type showed that treatment initiation prevalence was lower among both Medicaid and private insurance recipients aged 18–29 years (17% and 23%, respectively), compared with that among these recipients aged 50–59 years (28% and 42%, respectively) (Figure 2). Compared by insurance type, the odds of DAA treatment initiation were lowest among persons aged 18–29 and 30–39 years with Medicaid (aOR = 0.52 and 0.68, respectively) and among the same age groups for those with private insurance (0.42 and 0.62, respectively), and those aged 30–39 years with Medicare (0.56), compared with persons aged 50–59 years (Table 2).

**TABLE 2:** Adjusted odds* of initiation of direct-acting antiviral treatment of hepatitis C cases, by characteristic, insurance provider, and state Medicaid treatment restrictions — HealthVerity, United States, 2019–2020^†^

Characteristic	Multivariable aOR (95% CI)		
	Medicaid^§^	Medicare^¶^	Private
**Sex**			
Female	Ref	Ref	Ref
Male	0.85 (0.81–0.89)	0.79 (0.67–0.93)	0.90 (0.81–0.99)
**Age group, yrs**			
18–29	0.52 (0.49–0.57)	0.56 (0.21–1.50)	0.42 (0.35–0.51)
30–39	0.68 (0.64–0.73)	0.56 (0.39–0.88)	0.62 (0.53–0.73)
40–49	0.83 (0.77–0.89)	0.77 (0.56–1.07)	0.82 (0.70–0.96)
50–59	Ref	Ref	Ref
60–69	0.84 (0.79–0.91)	1.06 (0.87–1.28)	0.85 (0.80–0.90)
**Race**			
White	Ref	Ref	Ref
Black	0.93 (0.88–0.99)	1.03 (0.86–1.24)	1.08 (0.93–1.81)
Asian	0.99 (0.85–1.16)	1.56 (1.01–2.40)	1.30 (0.93–1.81)
Other	0.73 (0.62–0.88)	1.11 (0.67–1.89)	1.17 (0.95–1.46)
Missing	0.73 (0.67–0.79)	0.74 (0.57–0.96)	0.83 (0.73–0.95)
State Medicaid treatment restrictions**			
No	Ref	—	—
Yes^††^	0.77 (0.74–0.81)	—	—

**Abbreviations:** aOR = adjusted odds ratio; Ref = referent group.

* All models adjusted for sex, age group, and race. The Medicaid sample was also adjusted for Medicaid treatment restrictions. 95% CIs that exclude 1 were considered statistically significant.

^†^ Continuous enrollment in medical and pharmacy plans for ≥60 days before and ≥360 days after the RNA-positive index date during January 30, 2019–October 31, 2020.

^§^ Medicaid managed care.

^¶^ Medicare Advantage programs.

** Analysis restricted to Medicaid recipients only.

^††^ Living in a state with a Medicaid liver fibrosis or sobriety requirement (≥1 month of abstinence from alcohol or drugs) or prescriber restrictions.

Assessment of DAA treatment initiation by race and insurance type found that among Medicaid recipients, treatment initiation was lowest among persons of other races (20%) and those missing race information (19%) (Figure 2). Among private insurance recipients, treatment initiation was higher in all race groups, but was lowest among persons with missing race information (32%). In adjusted analyses, DAA treatment initiation was similar across most racial groups, except persons with missing race information, who had a lower prevalence of DAA treatment initiation relative to White persons for all insurance types (Table 2). In addition, both Medicaid recipients who reported Black or other race had lower prevalences of treatment initiation relative to White Medicaid recipients (aOR = 0.93 and 0.73, respectively); among Medicare recipients, Asian persons had higher rates of treatment initiation relative to White persons (aOR = 1.56). Male sex was consistently associated with lower treatment initiation among Medicaid, Medicare, and private insurance recipients (aOR = 0.85, 0.79, and 0.90, respectively).

In a model including variables for sex, age group, race, and insurance type, persons with hepatitis C with Medicaid and Medicare had lower odds of initiating DAA treatment than did those with private insurance (aOR = 0.54; 95% CI = 0.51–0.57 and aOR = 0.62; 95% CI = 0.56–0.68, respectively). Among Medicaid recipients, persons in states with Medicaid treatment restrictions had lower odds of receiving treatment than did those living in states without restrictions (aOR = 0.77).

## Discussion

Among adults aged 18–69 years with diagnosed HCV infection and continuous insurance coverage, approximately one third of those with private insurance and one quarter of Medicaid and Medicare recipients initiated DAA treatment within 360 days of diagnosis. Highly effective DAA treatment is recommended for persons with hepatitis C (*4*) and is curative in ≥95% of cases. Treatment saves lives, prevents ongoing transmission, and is cost saving (*5*–*8*), yet too few persons are receiving timely treatment (*8*,*12*–*14*), which could lead to both further progression of disease for the person infected with HCV as well as ongoing transmission to other persons.

Medicaid and Medicare recipients with hepatitis C were 46% and 38% less likely, respectively, to receive timely treatment compared with those with private insurance. Further, Medicaid recipients with diagnosed hepatitis C in states with Medicaid treatment restrictions were 23% less likely to receive timely treatment than were those living in states without restrictions. Medicare provides health insurance for persons aged ≥65 years living in the United States and persons with disabilities, and Medicaid provides health insurance for eligible adults and children in low-income households. Persons with low income experience social determinants of health that lead to negative health outcomes, including delays in timely treatment for health conditions (*14*). In general, Medicaid recipients have fewer financial resources and are more likely to be affected by social determinants of health, which further increases the likelihood of negative health outcomes associated with hepatitis C (*11*).

Although marketplace competition has reduced the net cost of DAAs, in 2014 initial costs for a course of all oral treatments exceeded $90,000, resulting in many insurers establishing restrictions to access (*14*). Current costs are considerably lower; however, Medicaid remains the least likely insurer to cover hepatitis C treatment. Treating all eligible patients without restriction would result in substantially reducing downstream negative clinical outcomes, decreasing the proportion of total costs attributable to future care, and producing considerable cost savings (14). Further, whereas hepatitis C treatment eligibility restrictions have become less stringent in some states, others maintain limitations on access to DAAs, including liver fibrosis qualifications, sobriety requirements, or medical subspecialist prescribing requirements. Removing these eligibility restrictions is necessary, but not sufficient. Addressing other barriers, including burdensome preauthorization requirements as well as integrating routine screening and treatment into primary care and other settings where persons with hepatitis C receive services, could also increase treatment coverage (*15*–*17*).

DAA treatment initiation was lowest among adults aged 18–29 and 30–39 years. These groups also have the highest rates of incident HCV infection, often in association with injection drug use, and the largest number of newly reported chronic infections (2). Early hepatitis C treatment prevents disease progression, limits future morbidity and mortality, and reduces health care costs by preventing cases of cirrhosis, liver transplantations, and hepatocellular carcinoma (*12*–*14*). Treatment of persons with ongoing transmission risk has important benefits beyond those to the person infected because with each successfully treated person, the number of persons able to transmit disease declines (*6*).

Medicaid recipients of other races were up to 27% less likely to initiate timely DAA treatment than White Medicaid recipients. The reasons for racial disparities in treatment initiation among continuously enrolled Medicaid recipients are unclear but might involve health system barriers associated with patient access, provider availability, quality of care, patient distrust, stigma, or language and cultural factors (*18*,*19*). The provision of culturally competent and timely hepatitis C treatment for racial and ethnic minority groups is essential to reducing existing disparities in hepatitis C–associated outcomes, including higher mortality among American Indian or Alaskan Native, Black, and Hispanic or Latino persons (8.63, 5.44, and 3.84 per 100,000 population, respectively) compared with that among White persons (3.08) (*2*,*11*,*16*,*19*).

Across insurance types, ≥75% of persons treated initiated treatment within 180 days after diagnosis. The smaller percentage of persons treated within 180 days after diagnosis might indicate lack of access to a hepatitis C treatment provider, insurance denial, or loss to follow-up. Treatment coverage can be increased by providing integrated care, patient navigation, and care coordination (*15*). The introduction of simplified hepatitis C treatment algorithms reducing the number of laboratory tests and in-person visits can facilitate patient-centered treatment (*20*).

The findings in this report are subject to at least five limitations. First, HealthVerity data might not be representative of DAA treatment patterns across the United States because of the sample characteristics of the payors and providers for whom they process data. Second, information on patients who are uninsured or incarcerated were not included; in addition, these data do not include persons who received care through the Veterans Health Administration. Third, the analytic cohort was conservatively defined, only including persons continuously enrolled for ≥60 days before and ≥360 days after the date of the positive index HCV RNA test result, which likely overestimates treatment initiation among all persons with hepatitis C HCV infection. Fourth, ethnicity data were missing for 61%, and race data for 13%, of the analytic cohort, which prevented examination of other potential treatment disparities. Finally, these data do not allow determination of whether absence of claims for treatment was the result of patient nonadherence, clinicians not prescribing DAAs, insurance providers not authorizing treatment, or prohibitive costs associated with copayments and deductibles. Further studies are needed to understand these barriers better.

Interventions to increase access to hepatitis C treatment with DAA agents include removing policies limiting patient eligibility based on fibrosis stage or sobriety, requiring treatment through specialists, and requirement for preauthorization (*11*,*17*). Universal hepatitis C screening coupled with simplified treatment protocols should be integrated into primary care and other settings serving persons with hepatitis C, and the number of primary care providers treating hepatitis C expanded, especially Medicaid providers serving populations disproportionately affected by hepatitis C. Increasing access to hepatitis C treatment to all populations, regardless of insurance type, is essential to reducing viral hepatitis–related disparities and achieving hepatitis C elimination.

**Summary**

**What is already known about this topic?**

Direct-acting antiviral (DAA) treatment is recommended for nearly all persons with hepatitis C and cures ≥95% of cases. Treatment saves lives, prevents transmission, and is cost saving.

**What is added by this report?**

Treatment rates are low overall and vary by age and insurance payor. DAA treatment is lowest among young adults aged 18–29 years and Medicaid recipients, and within Medicaid, among persons reporting Black or other race and persons in states with treatment restrictions.

**What are the implications for public health practice?**

Timely initiation of DAA treatment, regardless of insurance type, is critical to reducing viral hepatitis–related mortality, disparities, and transmission.

## Footnotes

* These authors contributed equally to this report.

† The January 2017–March 2020 estimate was obtained from the National Health and Nutrition Examination Survey (NHANES)(https://wwwn.cdc.gov/nchs/nhanes/continuousnhanes/default.aspx?cycle=2017-2020). The NHANES national probability sample includes the noninstitutionalized, civilian population of the United States; because it excludes certain populations known to have high hepatitis C prevalence from its sampling frame, NHANES underestimates the true prevalence of hepatitis C in the United States. During 2013–2016, researchers estimated that an additional 0.25 million persons in high-risk population groups unaccounted for by NHANES data were infected. https://www.cdc.gov/nchhstp/newsroom/2018/hepatitis-c-prevalence-estimates-press-release.html

§
https://healthverity.com/solutions/healthverity-marketplace/

¶
https://www.fda.gov/drugs/drug-approvals-and-databases/national-drug-code-directory

**
https://hepvu.org/hepatitis-c-treatment-restrictions-2/

†† 45 C.F.R. part 46, 21 C.F.R. part 56; 42 U.S.C. Sect. 241(d); 5 U.S.C. Sect. 552a; or 44 U.S.C. Sect. 3501 et seq.
